# Associations Among Various Physical Parameters and the Axillary‐to‐Rectal Temperature Difference in Dogs and Cats

**DOI:** 10.1111/vec.70020

**Published:** 2025-08-29

**Authors:** Cristian Martinez Alvarez, Britt A. L. Thevelein, Katie M. Hodges, Amy N. Weitzman, Amie Koenig, Benjamin M. Brainard

**Affiliations:** ^1^ Department of Small Animal Medicine and Surgery, College of Veterinary Medicine University of Georgia Athens Georgia USA; ^2^ Comp Sci Services Washington, DC USA

**Keywords:** axillary temperature, companion animal, hyperthermia, hypothermia, thermometry/thermometer

## Abstract

**Objective:**

To compare the measurement of axillary temperature (AT) with rectal temperature (RT) in dogs and cats and to identify the influence of physical parameters on the difference between their measurement using a veterinary‐specific thermometer.

**Design:**

Prospective study (2022–2024).

**Setting:**

University teaching hospital.

**Animals:**

A total of 106 dogs and 101 cats aged ≥4 months.

**Interventions:**

Body temperatures were measured contemporaneously using a veterinary‐specific, calibrated, dual‐thermistor axillary thermometer (AT) and a calibrated medical thermometer (RT). Data were evaluated for bias as a whole set and after stratification based on various physical parameters (species, body weight, haircoat length, body condition score [BCS]). A machine learning (ML) model was subsequently applied, and bias was reevaluated.

**Measurements and Main Results:**

A Bland–Altman analysis comparing the measurement of AT with RT showed a bias of 1.01°C (1.82°F) and 95% limits of agreement (LOA) −0.66°C to 2.68°C (−1.19°F to 4.83°F) in dogs and a bias of 0.23°C (0.42°F) and 95% LOA of −2.61°C to 3.08°C (−4.71°F to 5.55°F) in cats. Animals weighing <10 kg had a bias of 0.33°C (95% LOA: −2.29°C to 2.96°C), animals with BCS <5 had a bias of 0.09°C (95% LOA: −2.92°C to 3.11°C), and those with shorter haircoats had a bias of 0.49°C (95% LOA: −2.03°C to 3.00°C). The ML model overestimated RT when using AT and physical parameters in its algorithm (dogs: −0.57°C [95% LOA: −2.18°C to 0.99°C]; cats −0.84°C [95% LOA: −2.62°C to 0.93°C]).

**Conclusions:**

Animals weighing <10 kg, with lower BCS, and shorter haircoats had less bias when AT was compared with RT. Cats exhibited a lower bias between AT and RT than dogs. ML models can be programmed to account for various physical characteristics, improving the predictive impact of AT for directly measured RT, especially in categories of animals where closer prediction does not already exist.

**Abbreviations**
AT: axillary temperature
BCS: body condition score
BW: body weight
LOA: limits of agreement
ML: machine learning
RT: rectal temperature

## 1 Introduction

Accurately measuring body temperature is essential for diagnosing medical conditions and monitoring physiological responses [1, 2]. Although rectal temperature (RT) is the gold standard surrogate for measurement of core body temperature [3, 4], inaccurate readings can result from the presence of intestinal air, feces, or rectal trauma [1, 5, 6]. Some animals may also have conditions that make this technique less feasible (e.g., after perineal surgery). Furthermore, RT measurement can be stressful to the patient, which influences body temperature, and could pose a safety risk to the person taking the measurement or restraining the animal [1, 6].

Alternative, less invasive methods of temperature measurement that use traditional thermistors and infrared thermometers have shown variable accuracy when compared with measurement per rectum [5, 6, 7, 8, 9]. These less invasive techniques may be influenced by factors such as probe positioning, calibration, hypoperfusion, and the presence of hypothermia or hyperthermia, making them poor substitutes for RT. Newer thermometers with multiple thermistors have been developed with an aim of improving the accuracy of temperature measurement in companion animals. The measurement of body temperature using less invasive approaches and this newer technology has not yet been investigated [1, 6, 7, 10]. The further integration of advanced technologies with machine learning (ML) may result in developments that automatically take into account and adjust for the differences between newer techniques and the gold standard RT [1, 11].

Several studies have compared RT with axillary temperature (AT) but have not established that AT can serve as an accurate substitute for RT in dogs and cats [6, 12, 13]. Some of these reports have suggested that AT readings and, thus, accuracy of comparison may be influenced by various physical parameters [10, 14]. A clinically acceptable difference between RT and AT of <0.5°C has been previously proposed for the use of AT as a surrogate of RT in clinical practice [10], although evidence for the application of an across‐the‐board correction factor (e.g., adding 0.5°C to the measured AT to predict the RT) is likely an oversimplification of the relationship between AT and RT, which is complex and dependent on the physical characteristics of the animals in question.

In the current study, we used a novel dual‐thermistor thermometer to compare AT with RT. We hypothesized that differences in AT and RT measurements are influenced by body weight (BW), body condition score (BCS), and haircoat length. In addition, we hypothesized that ML could be developed to adjust for patient variability and create an algorithm to predict RT based on AT measurement.

## 2 Materials and Methods

This prospective study was performed from 2022 to 2024 and included data on dogs and cats in the anesthesia preparation room and emergency department of a university teaching hospital. Dogs and cats aged 4 months and older, of any sex and breed, were included in the study. Exclusion criteria were animals that were uncooperative, those with axillary or rectal trauma, individuals undergoing active cooling or heating, and measurements taken at ambient temperatures exceeding 25°C (77°F). All temperature measurements were obtained from animals that were housed for at least 3 h in a controlled setting where room temperature ranged between 22.0°C and 23.9°C (71.6°F–75°F). Measurements in anesthetized animals were obtained within 15 min of induction of anesthesia. Research protocols were reviewed by the institutional clinical research committee. Client consent was waived due to the noninvasive nature of the study and because of the accepted clinical practice of obtaining body temperature measurements per rectum as part of the in‐hospital management of the patients.

Recorded data included animal age, sex, species, breed, the ambient room temperature, AT and RT, systolic blood pressure (measured via Doppler ultrasonic flow probe), heart rate, respiratory rate, BW, BCS, and haircoat length. Ambient temperature measurements were obtained using a commercial hygrometer/thermometer^a^. ATs were measured using the small animal axillary probe of a calibrated, dual‐thermistor thermometer^b^ (measurement range: 25°C–45°C [77°F–113°F], with an accuracy of ±0.1°C [0.18°F]), and RTs were measured using a medical thermometer^c^ (measurement range: 26.7°C–43.3°C [80.06°F–109.94°F], with an accuracy of ±0.1°C [0.18°F]). Hypothermia and hyperthermia were defined as RT <37.5°C (99.5°F) and >39°C (102.5°F), respectively. ATs were acquired by placing the thermometer probe parallel to the humerus on the skin, with the forelimb resting on top of the thermometer with gentle pressure. The thermistor tip of the thermometer was placed in close proximity to the brachial plexus area. Acquisition of the AT was observed in real time using proprietary software^b^ with a computer connected to the thermometer via Bluetooth. The proprietary software for the AT measurement would not display a final temperature if the two thermistor measurements differed by more than 5%. During measurements, the software also provided a visual display of the temperature measured by both thermistors to allow visual comparison. The RT probe was covered by a proprietary probe sheath cover, and RT was obtained using the internal algorithm of the thermometer, which displayed a final single measurement. Data were recorded in both a computerized spreadsheet and a written record.

The BCS was assigned using a previously validated chart with scores ranging from 1 to 9 [15]. Lower BCS numbers refer to underconditioned individuals, compared with obese individuals, which are assigned higher numbers. A score of 5 was considered an ideal BCS. Haircoat was measured with a conventional rigid ruler and characterized as short when the average body hair length measured less than 2 cm, medium if length measured 2–2.5 cm, and long if the length exceeded 2.5 cm. If a physical characteristic was not recorded for a patient, it was excluded from the analysis for the pertinent variable.

### 2.1 Development of an ML Model

A supervised learning approach called a generative linear model was used in the current study, consisting of Bayesian ridge regression and written with the Python programming language and associated ML libraries. This method differs from linear regression analysis in that the ML model learns patterns and determines how the inputs affect the output by applying Bayesian logic to assess the likelihood of the inputs (i.e., AT and physical characteristics) occurring given the target output (i.e., RT). The dataset used in the initial training session consisted of 100 dogs and cats of various sizes and breeds and included the following features: AT, BW, breed size, BCS, haircoat length, ambient temperature, and sampling rate, which is the number of recorded temperature measurements collected by the probe. Sampling rate was included in the model because the training model generated a measurement every 230 ms, while the study set had a sampling rate of 300 ms. A function was written to convert the training set to a sampling rate of 300 ms to take this into account; hence, it was included in the model.

Training the model was the first phase of the ML process and began with preprocessing the data to standardize the inputs, including encoding categorical variables as discrete numeric values, scaling all the inputs on a scale of 0–1, calculating a data quality score based on how well the thermometer was calibrated and attached to the pet during the temperature reading, binning the temperature readings by averaging every 5 ms to remove routine anomalies, and normalizing the AT based on the point in time during the reading when the thermometer reached a plateau temperature, reflecting the final measured value. Low‐quality data were discarded from the training process. The preprocessed data were then split into a training set and a testing set, with 80% of the data for training and 20% for testing. The training data were fed into the model, which iteratively learned how the features relate and factor into deriving the target value to construct an underlying formula to predict the output based on the inputs.

After training, predictions were made by the model on the test set to determine the model's accuracy with inputs it did not see during the training phase. The accuracy of the training set versus the test set helps reveal whether the model is overfitting (where the model learns the features of the training data [and noise] too closely and cannot generalize to new inputs). The model hyperparameters, which are configuration parameters that control how the model is trained, such as how many iterations it goes through for learning, how many data points are analyzed at a time, and the function used to calculate the prediction error, were then tuned to improve the model performance and the model retrained as needed to reassess performance. Once an acceptable level of accuracy was achieved by the model as determined by Bland–Altman assessments of the bias between measurements of each iteration, the predictions were applied to the study dataset.

### 2.2 Statistical Analysis

Paired *t*‐tests were used to compare AT and RT readings from the same animal. Bland–Altman analyses were used to evaluate specific comparisons between AT and RT measurements for the general population and for specific subpopulations. The subpopulations considered for stratification of the data (i.e., evaluated separately) included species (dogs and cats), BW (<10 vs. >10 kg), BCS (<5, 5, and >5), and haircoat length (short, medium, and long). Descriptive statistics are displayed as mean ± SD for normally distributed data and median (minimum‐maximum) for nonparametrically distributed data.

## 3 Results

A total of 106 dogs and 101 cats were included in the analysis, with no animals being excluded; however, 11 animals did not have BCS recorded, and 5 did not have coat length recorded, and thus were excluded from relevant analyses. One hundred eighty‐five animals were assessed in the anesthesia preparatory area within 15 min of induction of anesthesia, and 22 animals were sampled in the emergency room. The median age of the dogs was 7 years (range: 4 months to 17 years), and the median age for cats was 2 years (range: 4 months to 19 years). The median BW was 20 kg for dogs (range: 1.15–70.76 kg) and 2.48 kg for cats (range: 1–8.5 kg). The median BCS was 5 (range: 1–9) for both species. Fourteen animals were considered to be long‐haired (6.76%), 33 medium‐haired (15.94%), and 155 short‐haired (74.88%).

There was a significant difference between mean RT (37.77 ± 1.36°C [99.98 ± 2.53°F]) and mean AT (36.58 ± 1.22°C [97.84 ± 2.56°F]; *p* < 0.001). The uncorrected RT–AT difference was within 0.5°C for 36 of 101 (36%) cats and 29 of 106 (27%) dogs. The mean temperature measurements for RT and AT in all the investigated categories of physical parameters can be found in Table 1. Bland–Altman analysis for all animals evaluating agreement between RT and AT calculated a bias of 0.63°C (1.14°F). When analyzing only dogs, a bias of 1.01°C (1.82°F) was found. Cats displayed a bias of 0.23°C (0.42°F).

**TABLE 1:** Mean ± SD, median, and range for contemporaneously measured AT and RT in 106 dogs and 101 cats.

Variables		*N*	Mean ± SD (AT) (°C)	Mean ± SD (RT) (°C)	AT median (min–max) (°C)	RT median (min–max) (°C)	*p*‐value (AT vs. RT)
Species	Dogs	106	36.90 ± 2.94	37.94 ± 0.90	37.00 (33.89–39.11)	37.89 (35.22–39.72)	<0.0001
	Cats	101	36.23 ± 4.56	36.47 ± 4.66	36.30 (33.27–39.17)	36.58 (32.83–39.55)	0.1093
BW	<10 kg	130	36.39 ± 4.42	36.73 ± 4.59	36.52 (33.27–39.17)	36.88 (36.94–39.72)	0.0523
	>10 kg	77	36.88 ± 2.80	38.00 ± 2.51	37.00 (34.83–39.11)	38.00 (34.33–39.55)	<0.0001
BCS^a^	<5	49	36.60 ± 4.62	36.70 ± 5.18	36.67 (33.28–39.11)	36.94 (32.83–39.56)	0.7532
	5	91	36.48 ± 4.08	37.09 ± 4.34	36.66 (33.44–39.17)	37.28 (33.17–39.33)	0.0017
	>5	56	36.78 ± 3.22	37.76 ± 3.15	36.89 (33.94–102.2)	37.89 (34.72–39.72)	<0.001
Haircoat length^a^	Short	155	36.50 ± 3.99	36.98 ± 4.43	36.66 (33.28–39.17)	37.22 (32.83–39.72)	0.0002
	Medium	33	36.78 ± 4.19	37.65 ± 4.15	37.17 (33.50–39.11)	37.72 (33.50–39.55)	0.0018
	Long	14	37.00 ± 3.06	38.17 ± 1.90	36.92 (35.50–38.17)	38.39 (36.78–38.89)	0.0004

Abbreviations: AT, axillary temperature; BCS, body condition score; BW, body weight; RT, rectal temperature.

^a^ Eleven animals did not have BCS recorded, and five animals did not have coat length recorded and were thus excluded from pertinent analyses.

Further stratifying the data, the bias in animals weighing <10 kg was 0.33°C (0.60°F; 95% limits of agreement [LOA]: −2.29°C to 2.96°C [−4.12°F to 5.33°F]), and those weighing >10 kg had a bias of 1.11°C ([2.00°F], 95% LOA: −0.62°C to 2.85°C [−1.12°F to 5.14°F]). Because the majority (101/130) of animals weighing <10 kg were cats, Bland–Altman analysis was performed for each individual species within the group of animals weighing <10 kg. With this stratification, cats displayed a bias of 0.25°C (0.45°F; 95% LOA: −2.65°C to 3.14°C [−4.80°F to 5.65 °F]), while the dogs showed a bias of 0.49°C (0.88°F; 95% LOA: −1.59°C to 2.57°C [−2.87°F to 4.63°F]). Bias in animals with a BCS of <5 was 0.09°C (0.17°F; 95% LOA: −2.92°C to 3.11°C [−5.26°F to 5.60°F]), while for those animals with a BCS of 5, it was 0.61°C (1.10°F; 95% LOA: −1.63°C to 2.85°C [−2.94°F to 5.14°F]), and for animals with a BCS of >5, the bias was 0.97°C (1.75°F; 95% LOA: −0.78°C to 2.72°C [−1.40°F to 4.90°F]). The raw data indicated that 18 of 49 (37%) animals with a BCS of <5, 27 of 91 (30%) animals with a BCS of 5, and 16 of 56 (29%) animals with a BCS of >5 had RT‐to‐AT differences within 0.5°C. Animals with shorter haircoats exhibited a bias of 0.49°C (0.88°F; 95% LOA: −2.03°C to 3.00°C [−3.66°F to 5.40°F]) compared with those with medium (0.87°C [1.56°F]; 95% LOA: −1.02°C to 2.75°C [−1.84°F to 4.96°F]) and long haircoats (1.16°C [2.09°F]; 95% LOA: −0.68°C to 3.00°C [−1.22°F to 5.40°F]).

**FIGURE 1:**
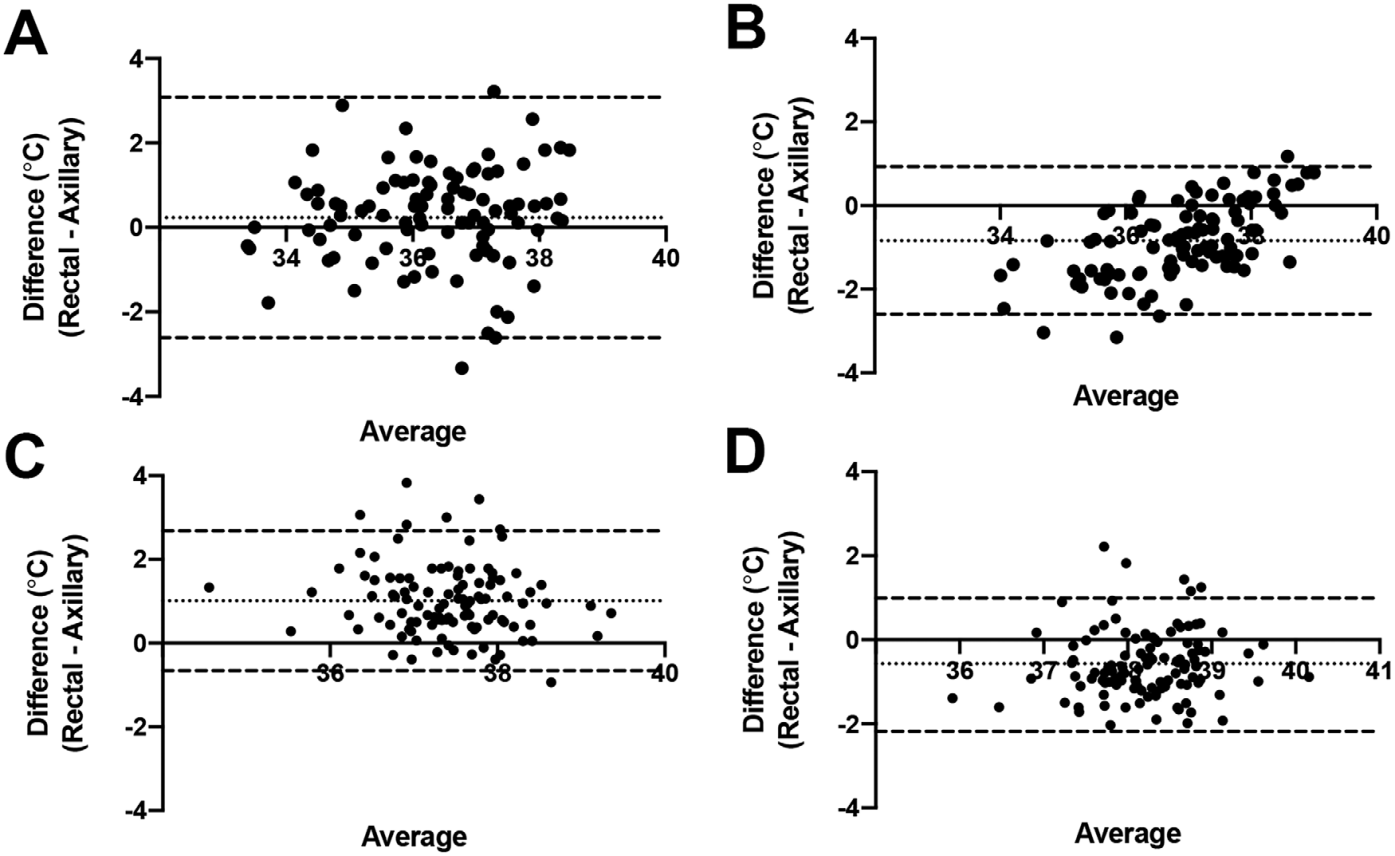
Bland–Altman analysis for dogs and cats before and after applying the predictive algorithm. (A) Analysis for cats before prediction. (B) Analysis for cats after prediction. (C) Analysis for dogs before prediction. (D) Analysis for dogs after prediction.

The ML model was applied to the data collected from the AT measurements in the current study. No additional training of the model was provided beyond what was previously described, and AT was compared with the measured RT. The previously mentioned ML was trained as described and used on the current dataset to predict the RT using AT measurements and the recorded patient features (e.g., BW, BCS, and haircoat length). Collectively, the estimated thermometer readings across all data overestimated the measured RT, with a bias of −0.72°C (−1.28°F; 95% LOA: −2.40°C to 0.97°C [−4.32°F to 1.75°F]). Dogs and cats analyzed separately both demonstrated biases similar to the combined group (Figure 1). Dogs exhibited a bias of −0.57°C (−1.08°F; 95% LOA: −2.18°C to 0.99°C [−3.92°F to 1.80°F]), and cats displayed a bias of −0.84°C (−1.08°F; 95% LOA: −2.62°C to 0.93°C [−4.70°F to 1.67°F]).

## 4 Discussion

In the current study, AT was an acceptable surrogate for RT in cats; however, this was not the case in dogs. This study also found that physical parameters (BW, BCS, and haircoat length) had a significant influence on AT. Specifically, higher BW, higher BCS, and longer haircoats worsened the bias between AT and RT, suggesting that these body characteristics reduce the ability for AT to predict RT. Although ML overestimated RT in dogs, it was useful in reducing bias between AT and RT when physical parameters were put into the algorithm.

Regardless of patient characteristics, AT always read lower than RT, and there is a concern that this might potentially mask the diagnosis of elevated RT. Unfortunately, there were no dogs in this cohort with an RT above 39.5°C (103.1°F) and few with RT >39.17°C (102.5°F), so conclusions on the impact of hyperthermia on the relative AT reading cannot be made. The agreement between AT and RT in previous studies is variable, with some claiming good agreement [6] and others arguing that AT is not an acceptable substitute for RT [7, 12, 13, 14, 16]. It is important to note that some of these previous studies did not stratify pet features and evaluated a more diverse population as a whole. A previous study in 150 cats found that the difference between AT and RT was greater as measured RT increased; therefore, the possibility that the association between AT and RT is not a linear function should be considered [10].

AT readings of cats in the current study exhibited an acceptable bias when compared with RT measurements. This may be because the studied cats were smaller with a lower BCS and shorter haircoats compared with the dogs. In practical terms, AT measurements may be particularly useful in cats with this body type, especially when they are not amenable to rectal measurements. The results presented here are consistent with a previous report in which AT in cats had better agreement with RT when compared with dogs. Similar to our study, this report also found that high BCS negatively influenced the agreement between AT and RT. It is interesting to note that the authors found worse agreement between AT and RT when looking at neuter status and between the left and right axilla [10]. Unfortunately, in the current study, we only evaluated neutered cats and did not record the side of temperature measurement.

When comparing AT with RT in dogs, our study is in agreement with others showing that AT is an inadequate direct surrogate for RT. Mathis and Campbell [13] investigated the association of AT and RT using healthy Beagle dogs under controlled ambient conditions and found poor correlation between AT and RT, which would have been expected to be greater given the specific physical characteristics of the studied dogs. Cugmas et al. [7] also found a very poor correlation between AT and RT when using a custom‐made infrared thermometer in 204 healthy dogs, which identified the inguinal region as having the best correlation with RT.

Lamb et al. [14] studied the influence of BW, BCS, and haircoat in 212 healthy dogs. Although they found a better agreement between AT and RT in dogs compared with the current study, their bias of 0.6°C does not fall within the acceptable bias of 0.5°C as defined a priori. When evaluating pet features, they found that animals with a higher BCS had better agreement between AT and RT than animals with a normal or low BCS. Additionally, that study found better agreement between AT and RT in animals with a longer haircoat compared with short or medium haircoats. These findings on BCS and haircoat are in opposition to our study, which found greater bias at a higher BCS and with longer haircoats.

Adipose tissue, which accumulates in patients with a higher BCS, has a low thermal conductivity. Larger sized dogs, even of normal BCS, may also have more tissue between the axilla and the core body. This may explain why the temperature difference between AT and RT became more pronounced as weight increased. Although the BCS results were not surprising, the haircoat data were somewhat unexpected, as bias increased from shorter to longer haircoat length. Because the axillary thermometer was placed in an area of relatively hairless skin, it was not anticipated that the bias of measurement would vary to the described degree once dogs were stratified. Coat length and thickness may affect the temperature of the periphery by modulating the interaction of airflow with the bare skin; however, in the controlled setting of a veterinary hospital, it is unclear why this may have made a difference. Comparatively, the evaluation group comprised many fewer animals with medium or long hair, and additional work focused on expanding this sample group and further characterizing or distinguishing dogs with a thick double coat may address the variability among animals with different haircoat lengths.

The ML algorithm used in the current study to predict the association of RT with AT differed from the methodology of the previously mentioned study [14], which used linear and multivariate regression analysis to predict RT based on AT. Regression analysis assumes fixed relationships between the independent and dependent variables, and a single equation may be unable to capture complex relationships between multiple independent variables. Because the relationship between AT and RT measurements depends on various characteristics that have a more complex relationship, this study attempted to improve upon regression methods by applying ML. ML involves developing algorithms that can autonomously produce outputs by identifying underlying patterns in the inputs that are often imperceptible or infeasible for people to calculate. Supervised learning is a type of ML where the model is trained on labeled data (i.e., including both the independent and dependent variable values) so that it can assess the accuracy of its predictions and improve as the algorithm is adjusted. Using an ML approach enabled the creation of a mathematic function to predict the RT, considering the underlying relationships between the inputs that cannot easily and accurately be captured by a linear or log‐linear function.

Prediction of the RT based on the measured AT using the ML model resulted in an overestimation of the measured RT, but the range of temperature values within the LOA was narrower compared with that of the nonpredicted values. Previous publications have recommended a difference between AT and RT of <0.5°C (0.9°F) for clinical use [10]. Although the model approached this difference, it did not achieve this criterion. Further training of the model with greater numbers of animals with more variability in physical parameters could improve its predictive capabilities. Notably, the application of the ML model to cats actually increased the bias. We suspect that this may be related to the relative contributors of BW in cats versus dogs. While larger breed dogs are heavier partially due to bone structure, cats have a relatively limited size range, so differences in weight are more likely to be from changes in BCS. This difference between species suggests that specific ML models should be developed for cats versus dogs and that one model cannot be used across the board.

As expected, all cats were included in the <10 kg group, which would overrepresent the number of smaller animals in the current study. Additionally, by including cats and dogs in the same group regarding other weight parameters, such as BCS, we did not stratify the effect of weight on temperature variations in cats. Our data correspond with previous reports that increasing BCS likely affects the difference between AT and RT measurements.

A protocol‐specific reference interval for AT can be generated in practices using a standard measurement protocol for AT, potentially identifying patients with increased RT; however, this assumes a linear difference between AT and RT and ignores the influence of BCS and animal size, as documented in the current study.

Several limitations of our study should be acknowledged. Many of the animals in the study were normothermic or hypothermic, which was likely a result of the inclusion of many animals in the perianesthetic period. This resulted in an underrepresentation of hyperthermic animals. Furthermore, our population included a few animals that presented as emergencies, but the impact of shock or perfusion disorders on AT or RT was not evaluated. Likewise, the impact of age‐related changes on vascular compliance was not assessed. We also acknowledge that perfusion changes as a result of anesthetic drugs (e.g., vasodilation from propofol administration) or underlying patient pathology were not directly assessed. Other aspects of the study population that may have led to bias in the measurements were the relatively low number of very large dogs and of animals classified as long haired. Additionally, the use of different thermometers for AT and RT measurements could introduce variability, although we intentionally used a calibrated small animal axillary thermometer and an advanced medical thermometer to enhance reliability of measurement.

This study found good agreement between AT and RT in cats, suggesting that AT may be used in clinical practice as a surrogate for RT. We confirmed that specific physical parameters such as BW, BCS, and haircoat may influence the relationship between AT and RT, and the impact of these parameters must be assessed before clinical use of AT to predict RT, especially because the bias is not predictable enough to have a single correction factor that can be applied to AT to predict RT. Future studies should encompass a larger and more diverse population of animals. Because the majority of the animals in this study were normothermic, the influence of hypothermia or hyperthermia on the bias between AT and RT remains unclear and should be a focus of future studies. In animals with significant perfusion abnormalities or hyperthermia or hypothermia, direct measurement of RT is likely the optimal way to accurately estimate core body temperature. Intermittent comparisons of AT and RT in a specific animal with stable perfusion parameters may allow predictions of RT from measured AT for that individual animal and, thus, clinical use of the AT for temperature monitoring. If ML models can be validated to take phenotypic variability into account to predict RT from AT measurements, these caveats may become less critical.

## Author Contributions

**Cristian Martinez Alvarez**: data curation, investigation, writing – original draft, writing – review and editing. **Britt A. L. Thevelein**: conceptualization, writing – review and editing. **Katie M. Hodges**: data curation. **Amy N. Weitzman**: formal analysis, methodology, writing – review and editing. **Amie Koenig**: writing – review and editing. **Benjamin M. Brainard**: conceptualization, funding acquisition, methodology, supervision, writing – original draft, writing – review and editing.

## Conflicts of Interest

Dr. Brainard is an Associate Editor of the Journal but only participated in the peer review process as an author. The authors declare no other conflicts of interest.

## Endnotes

a AcuRite 00613 Digital Hygrometer & Indoor Thermometer, AcuRite, Lake Geneva, WI.

b Mella Pro, Mella Pet Care Inc., Chicago, IL.

c Welch Allyn 692, Welch Allyn, Inc, Skaneateles Falls, NY.
